# Case Report: Diagnostic uncertainty in IC-MPGN coexisting with IgM-κ MGUS

**DOI:** 10.3389/fmed.2026.1869336

**Published:** 2026-07-08

**Authors:** Qishun Wu, Ling Yang, Xin Lin, Zhiliang Yu, Zhangli Wu

**Affiliations:** Department of Nephrology, The Second Affiliated Hospital of Wannan Medical University, Wuhu, Anhui, China

**Keywords:** diagnostic uncertainty, IgM-k monoclonal gammopathy, immune complex-mediated membranoproliferative glomerulonephritis, monoclonal gammopathy of renal significance, monoclonal gammopathy of undetermined significance, rituximab

## Abstract

**Background:**

Distinguishing immune complex-mediated membranoproliferative glomerulonephritis (IC-MPGN) with coincident monoclonal gammopathy of undetermined significance (MGUS) from monoclonal gammopathy of renal significance (MGRS) remains challenging when biopsy sampling is inadequate and gold-standard pathologic workup is incomplete.

**Case Presentation:**

A 60-year-old woman presented with upper abdominal fullness and heartburn. Routine testing revealed nephrotic-range proteinuria, acute kidney injury (AKI), and hypocomplementemia. Serum immunofixation showed IgM-κ monoclonal gammopathy. Bone marrow examination identified a clonal B-lymphocyte population of approximately 1% with no evidence of lymphoplasmacytic lymphoma or multiple myeloma. Kidney biopsy demonstrated IC-MPGN type I with mesangial and subendothelial electron-dense deposits. Immunofluorescence revealed granular deposition of IgG, IgM, C3, kappa, and lambda, indicating a polyclonal immune complex pattern. Cryoglobulin testing was negative on three occasions under strict 37 °C conditions. These findings suggested but could not confirm IC-MPGN with coincident IgM-κ MGUS; MGRS could not be excluded because only one non-sclerotic glomerulus was available for light microscopy and pronase-digested paraffin immunofluorescence was not performed. The patient received methylprednisolone pulse therapy followed by rituximab. At 1-month follow-up, serum albumin increased from 25.6 to 31.3 g/L, serum creatinine decreased from a peak of 231.0 to 94.0 μmol/L, and 24-h urine protein decreased from 7,930 mg to 1.1 g. Complement C3 and C4 normalized. Clinical improvement was observed following combined therapy; however, the independent contribution of rituximab cannot be determined from this single case.

**Conclusion:**

This case underscores the diagnostic uncertainty inherent in distinguishing coincident MGUS from MGRS when polyclonal immunofluorescence coexists with monoclonal gammopathy but gold-standard pathologic workup is incomplete. It highlights the need for standardized pathologic evaluation, including pronase-digested paraffin immunofluorescence, and illustrates that routine urinalysis in older adults with non-renal symptoms can reveal clinically significant kidney disease.

## Introduction

Membranoproliferative glomerulonephritis (MPGN) is a histopathological pattern characterized by mesangial hypercellularity, endocapillary proliferation, and double-contour formation of the glomerular basement membrane (1, 2). Based on immunopathological features, MPGN is classified into immune complex-mediated MPGN (IC-MPGN) and complement-mediated MPGN (C3 glomerulopathy) 0.1 IC-MPGN involves deposition of circulating immune complexes with subsequent complement activation leading to progressive kidney injury (1, 2).

Monoclonal gammopathy of undetermined significance (MGUS) is a clonal proliferation of plasma cells or B lymphocytes. When monoclonal immunoglobulin causes kidney damage directly or indirectly, the condition is termed monoclonal gammopathy of renal significance (MGRS) 0.3 (4). MGRS encompasses diverse glomerular lesions, including proliferative glomerulonephritis with monoclonal immunoglobulin deposits (PGNMID), which typically demonstrates MPGN-like lesions with monotypic light chain restriction, most commonly involving IgG3 with restricted kappa or lambda expression (5, 6).

The coexistence of IC-MPGN and monoclonal immunoglobulin is uncommon. Distinguishing coincident MGUS from true MGRS carries therapeutic implications: PGNMID often requires clone-directed therapy, whereas IC-MPGN may respond to immunosuppression targeting immune complex-mediated inflammation (7, 8). This report presents a diagnostically challenging case in which polyclonal immunofluorescence findings on eight glomeruli coexisted with a monoclonal gammopathy, yet definitive classification remained limited by tissue sampling constraints. The case contributes to the literature by illustrating the specific diagnostic gray zone where comprehensive but incomplete pathologic workup cannot resolve the MGUS vs. MGRS distinction—a scenario that is underrepresented in existing case series and carries important therapeutic implications. The central teaching point is that, in the absence of gold-standard pathologic workup, clinicians must make treatment decisions recognizing that the classification remains provisional.

### Case presentation

A 60-year-old woman presented with upper abdominal fullness and heartburn for 4 days and was admitted to the gastroenterology department on March 24, 2026. Her past medical history included cerebral infarction and essential hypertension. On admission, blood pressure was 148/92 mmHg, heart rate 86 beats/min, respiratory rate 18 breaths/min, and temperature 36.5 °C. Physical examination revealed no peripheral edema, ascites, or signs of volume overload. She was admitted with a working diagnosis of acute gastritis. The clinical timeline is summarized in Table 1.

**Table 1:** Clinical timeline.

Date	Event	Key findings	Treatment
March 24, 2026	Admission to gastroenterology	Upper abdominal fullness, heartburn, nausea	–
March 25, 2026	Transfer to nephrology	Proteinuria 4+, hematuria 3+, creatinine 143.0 μmol/L, eGFR 34.3 ml/min/1.73 m^2^	–
March 26, 2026	Laboratory workup completed	Severe hypoalbuminemia, hypocomplementemia, elevated IgM, elevated free κ light chain	–
March 30, 2026	Kidney biopsy performed; methylprednisolone initiated	Biopsy sample limited (one non-sclerotic glomerulus on LM); mesangial and subendothelial deposits; pseudothrombi noted	Methylprednisolone 500 mg IV × 3 days
April 2, 2026	Pathology report finalized	IC-MPGN type I; IgM 2+, kappa 1+, lambda 2+; polyclonal pattern; cryoglobulinemia suggested by pathologist	–
April 3, 2026	Rituximab administered	First dose 500 mg IV	Rituximab 375 mg/m^2^ (~500 mg) IV
April 4, 2026	Serum immunofixation result; first cryoglobulin test	IgM-κ monoclonal gammopathy confirmed; cryoglobulin negative (37 °C processing)	–
April 7, 2026	Bone marrow examination (sampled)	Aspirate and biopsy: no lymphoplasmacytic lymphoma or myeloma; ~1% clonal B cells by flow; plasma cells < 5%	–
April 7, 2026	Discharge	Edema improved; creatinine peaked at 231.0 μmol/L, eGFR nadir 19.16 ml/min/1.73 m^2^	–
April 14, 2026	Second cryoglobulin test	Negative (37 °C processing)	–
April 28, 2026	1-month follow-up; third cryoglobulin test; second rituximab dose	Edema resolved, appetite recovered; albumin 31.3 g/L; creatinine 94.0 μmol/L; eGFR 56.81 ml/min/1.73 m^2^; 24 h urine protein 1.1 g; C3/C4 normalized; free light chains and M protein modestly declined; cryoglobulin negative	Rituximab 375 mg/m^2^ (~500 mg) IV (2nd dose)

Given the gastrointestinal symptoms, an abdominal ultrasound was performed on admission, which showed no gallstones, biliary dilation, or pancreatic abnormalities. The kidneys appeared normal in size and echogenicity. Upper gastrointestinal endoscopy was not performed because the patient's symptoms improved with proton pump inhibitor therapy and the laboratory abnormalities (proteinuria, hematuria, elevated creatinine) prompted nephrology consultation before endoscopic evaluation could be arranged.

Admission laboratory testing revealed AKI and nephrotic syndrome (Table 2). Serum creatinine was 143.0 μmol/L with an estimated glomerular filtration rate (eGFR) of 34.3 ml/min/1.73 m^2^. Urinalysis showed protein (4+) and occult blood (3+). The 24-h urine protein was 7,930 mg. Serum albumin was 25.6 g/L. Complement C3 was low at 0.59 g/L (reference 0.9–1.8 g/L), and complement C4 was at the lower limit of normal at 0.10 g/L (reference 0.1–0.4 g/L). Immunoglobulin levels showed low IgG (3.96 g/L) and elevated IgM (2.63 g/L). Serum free light chain assay revealed markedly elevated free kappa light chains (323.13 mg/L; reference 3.30–19.40 mg/L) and mildly elevated free lambda light chains (28.79 mg/L; reference 5.71–26.30 mg/L), with a kappa/lambda ratio of 11.224 (reference 0.26–1.65).

**Table 2:** Key laboratory findings at admission and follow-up.

Parameter	Admission	Peak/nadir	1-month follow-up	Reference range
Serum creatinine (μmol/L)	143.0 (peak 231.0)	231	94.0	44–97
Blood urea nitrogen (mmol/L)	8.87	–	6.7	2.6–7.5
Serum albumin (g/L)	25.6	–	31.3	40–55
Total protein (g/L)	46.2	–	63.2	65–85
Serum calcium (mmol/L)	1.96	–	2.21	2.11–2.52
Serum potassium (mmol/L)	3.30	–	3.6	3.5–5.3
Complement C3 (g/L)	0.59	–	Normalized	0.9–1.8
Complement C4 (g/L)	0.10	–	Normalized	0.1–0.4
Immunoglobulin G (g/L)	3.96	–	9.3	7.0–16.0
Immunoglobulin M (g/L)	2.63	–	2.2	0.4–2.3
C-reactive protein (mg/L)	25.20	–	8.1	< 10
24-h urine protein (mg/24h)	7,930.40	–	1,100	< 150
Urine protein dipstick	4+	–	+	Negative
Urine occult blood	3+	–	+	Negative
Free κ light chain (mg/L)	323.13	–	267.50	3.30–19.40
Free λ light chain (mg/L)	28.79		24.30	5.71–26.30
Free κ/λ ratio	11.224	–	11.008	0.26–1.65

^*^Normalized values at follow-up.

Serum protein electrophoresis demonstrated an M protein spike of 2.7% in the gamma region. Serum immunofixation electrophoresis confirmed an IgM-κ monoclonal gammopathy. Bone marrow aspirate and biopsy performed on April 7, 2026, showed no morphological evidence of lymphoplasmacytic lymphoma or multiple myeloma. Flow cytometry identified an abnormal B-lymphocyte population comprising approximately 1% of nucleated cells, with the phenotype CD5-negative, CD10-negative, HLA-DR-positive, CD19 (bright), CD20 (bright), CD22-positive, CD79b-positive, FMC7-positive, and surface kappa-positive. Bone marrow plasma cells accounted for less than 5% of nucleated cells. These findings supported a diagnosis of MGUS according to International Myeloma Working Group criteria. MYD88 L265P mutation testing was not performed, as serum IgM level (2.63 g/L) was below the typical threshold for Waldenström macrogulinemia (>3 g/L) and there were no clinical features of hyperviscosity.

Infectious and autoimmune workup was negative for hepatitis B surface antigen, hepatitis C antibody, hepatitis C RNA, and HIV antibody. Antinuclear antibody, anti-double-stranded DNA, and anti-neutrophil cytoplasmic antibody were negative. Rheumatoid factor was not measured.

The patient was transferred to nephrology on March 25, 2026. A percutaneous kidney biopsy was performed on March 30, 2026. The biopsy sample was limited: light microscopy evaluated only one non-sclerotic glomerulus (two additional glomeruli were globally sclerosed). Immunofluorescence examined eight glomeruli, and electron microscopy examined two glomeruli. This sampling constraint may have affected the representativeness of pathological findings and the ability to detect focal monotypic deposits.

Light microscopy showed that the remaining glomerulus demonstrated moderate-to-severe mesangial and endocapillary hypercellularity with inflammatory cell infiltration. Periodic acid-methenamine silver staining revealed mesangial interposition and double-contour formation of the glomerular basement membrane. Eosinophilic deposits were present in the mesangial area and subendothelium. Some capillary lumens contained pseudothrombi. Tubular epithelial cells showed vacuolar degeneration, with focal tubular atrophy affecting approximately 10% of the cortical area. The interstitium showed multifocal inflammatory cell infiltration with fibrosis.

Immunofluorescence revealed IgG (1+), IgM (2+), C3 (+/−), kappa (1+), lambda (2+), and C4d (1+) along capillary walls and mesangial areas in a granular, discontinuous pattern. IgA, C1q, and fibrinogen were negative. The presence of both kappa and lambda light chains was interpreted as a polyclonal immune complex deposition pattern rather than monotypic immunoglobulin restriction. The intensity difference (kappa 1+ vs. lambda 2+) was considered non-specific and segmental.

Electron microscopy showed partial capillary loop collapse with luminal narrowing. Diffuse podocyte foot process effacement was present. Mesangial and endothelial cells were markedly proliferated, with mesangial interposition causing double-contour changes. Electron-dense deposits were identified in the mesangial area and subendothelium. No organized deposits, microtubular structures, fibrillar formations, fingerprint-like structures, or crystalline structures were observed.

**Figure 1:**
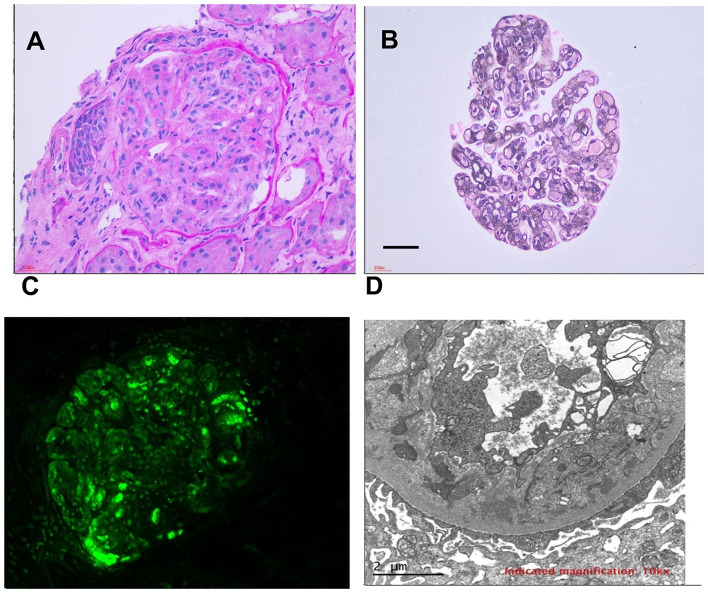
Kidney biopsy findings. Kidney biopsy findings in immune complex-mediated membranoproliferative glomerulonephritis type I coexisting with IgM-kappa monoclonal gammopathy of undetermined significance. **(A)** Periodic acid-Schiff staining (×400, scale bar = 50 μm) showing mesangial hypercellularity and endocapillary proliferation. **(B)** Periodic acid-methenamine silver staining (×400, scale bar = 50 μm) demonstrating mesangial interposition and double-contour formation of the glomerular basement membrane. **(C)** Immunofluorescence for IgM (×400, scale bar = 50 μm) showing granular deposits along capillary walls and mesangial areas; note that both kappa (1+) and lambda (2+) light chains were positive, indicating a polyclonal pattern. **(D)** Electron microscopy showing subendothelial electron-dense deposits (arrowheads), mesangial interposition causing double-contour changes, and diffuse podocyte foot process effacement. No organized microtubular structures, fibrillar formations, or fingerprint-like structures were observed. Scale bars: 50 μm **(A–C)**; 2 μm **(D)**. All photomicrographs were obtained from the same limited biopsy sample (one non-sclerotic glomerulus on light microscopy).

The final pathological diagnosis was immune complex-mediated membranoproliferative glomerulonephritis (type I MPGN on electron microscopy) (Figure 1). The pathologist noted that the presence of pseudothrombi warranted consideration of cryoglobulinemic glomerulonephritis.

Following the pathology report (finalized April 2, 2026), serum immunofixation (April 4, 2026) and bone marrow examination (sampled April 7, 2026) confirmed IgM-κ MGUS. The patient did not meet criteria for Waldenström macrogulinemia, multiple myeloma, or lymphoplasmacytic lymphoma.

Cryoglobulin testing was performed on three occasions (April 4, April 14, and April 28, 2026) under strict 37 °C conditions from collection through centrifugation, followed by 7-day incubation at 4 °C with daily inspection. No cryoprecipitate was observed on any occasion. The repeatedly negative results, combined with negative hepatitis C RNA, absence of extrarenal manifestations (purpura, arthralgia, neuropathy, Raynaud phenomenon), and the lack of organized microtubular structures on electron microscopy, made cryoglobulinemic glomerulonephritis unlikely.

The working diagnosis at discharge was IC-MPGN type I with coincident IgM-κ MGUS. However, definitive classification was not possible: MGRS could not be excluded because of limited biopsy sampling (only one non-sclerotic glomerulus on light microscopy) and the absence of pronase-digested paraffin immunofluorescence, a technique that might unmask hidden monotypic deposits and which represents a diagnostic gold standard not performed in this case. We have retrospectively confirmed that no additional paraffin blocks or frozen tissue remain available for this procedure. Consequently, the distinction between coincident MGUS and true MGRS cannot be resolved in this patient.

The patient received intravenous methylprednisolone 500 mg daily for three consecutive days starting March 30, 2026. Given the AKI and nephrotic-range proteinuria, corticosteroids were initiated promptly to suppress immune complex-mediated inflammation. Rituximab was subsequently administered at 375 mg/m^2^ (approximately 500 mg for this patient, body surface area 1.58 m^2^) on April 3, 2026, as part of a four-dose weekly induction regimen. This dosing follows the standard lymphoma protocol. The optimal dosing for IC-MPGN with coincident MGUS remains undefined. At the time of writing, the induction course was ongoing and peripheral blood CD19/CD20 B-cell counts had not yet been monitored. Given the concurrent administration of high-dose corticosteroids and the absence of B-cell monitoring data, the observed clinical improvement cannot be attributed to rituximab independently. The patient received no Pneumocystis jirovecii pneumonia prophylaxis, a limitation given the immunosuppressive intensity.

Supportive care included proton pump inhibitor therapy, diuretics, and fluid management. Angiotensin-converting enzyme inhibitor or angiotensin receptor blocker therapy was deferred due to AKI and hyperkalemia risk.

The patient was discharged on April 7, 2026. At 1-month follow-up (April 28, 2026), edema had completely resolved and appetite had recovered. Laboratory reassessment showed serum albumin 31.3 g/L, serum creatinine 94.0 μmol/L (down from a peak of 231.0 μmol/L), eGFR 56.81 ml/min/1.73 m^2^ (up from a nadir of 19.16 ml/min/1.73 m^2^), and 24-h urine protein 1.1 g. Complement C3 and C4 had normalized. Serum free light chains decreased from 323.13 to 267.50 mg/L for kappa and from 28.79 to 24.30 mg/L for lambda, with the kappa/lambda ratio showing no significant change (11.224 to 11.008). M protein remained detectable at 2.5%. A second intravenous dose of rituximab was administered on April 28, 2026. The patient remains under ongoing nephrology and hematology surveillance with planned reassessment at 3-month intervals. Long-term outcomes regarding sustained remission and clone evolution remain unknown.

## Discussion

This case illustrates a 60-year-old woman with type I IC-MPGN and IgM-κ MGUS in whom the renal disease was incidentally discovered during evaluation for nonspecific gastrointestinal symptoms. Most reported cases of MGRS-associated glomerulonephritis present with proteinuria, hematuria, or edema (3). Our patient's initial presentation underscores the value of routine urinalysis and kidney function assessment in older adults with non-renal symptoms. The differential diagnosis is summarized in Table 3.

**Table 3:** Differential diagnosis: IC-MPGN with MGUS vs. PGNMID vs. cryoglobulinemic glomerulonephritis.

Feature	This case (IC-MPGN + MGUS)	PGNMID	Cryoglobulinemic GN	This case: conclusion
Clinical presentation	**Nephritic syndrome with nephrotic-range proteinuria, AKI**	Nephrotic syndrome, hematuria	Nephrotic syndrome, purpura, arthralgia, neuropathy	Consistent with IC-MPGN
Serum monoclonal protein	IgM-κ MGUS (~1% clone)	IgG3-κ most common; other IgG subclasses and IgM/A possible	Type II: monoclonal IgM with RF activity	Consistent with MGUS
Complement profile	Low C3, low-normal C4	Variable	Low C3, low C4 (classical pathway)	Consistent with IC-MPGN
Immunofluorescence	IgG+, IgM+, C3+, κ+, λ+ (polyclonal)	Monotypic IgG (commonly IgG3) with κ or λ restriction	IgM, C3; κ or λ restriction in type II	Argues against PGNMID and cryoglobulinemia
Electron microscopy	Subendothelial and mesangial deposits; no organized structures	Subendothelial, mesangial, subepithelial deposits	Subendothelial deposits, pseudothrombi, organized microtubules (when present)	Consistent with IC-MPGN
Cryoglobulin testing	Negative (three occasions, 37 °C processing)	Not applicable	Typically positive	Argues against cryoglobulinemia
Key diagnostic features	Polyclonal IF pattern; negative cryoglobulins	Monotypic deposits on IF	Serum cryoglobulins; organized microtubules on EM	Supports IC-MPGN with coincident MGUS
Diagnostic certainty in this case	Highly suggestive but not definitive	Not supported by available IF, but not definitively excluded because of limited sampling and lack of paraffin IF	Unlikely (repeatedly negative cryoglobulins, negative HCV, no extrarenal features)	–
Therapeutic approach	Immunosuppression + clone-directed therapy if MGRS confirmed	Clone-directed therapy (rituximab, bortezomib)	Immunosuppression, plasmapheresis, rituximab	Empirical combined approach
Recommended distinguishing test	Paraffin IF with pronase digestion	Paraffin IF with pronase digestion	Serum cryoglobulin testing under strict 37 °C conditions	Not performed (tissue unavailable)

### Diagnostic uncertainty and limitations

The central diagnostic challenge was distinguishing IC-MPGN with coincident MGUS from true MGRS, specifically PGNMID. PGNMID typically demonstrates MPGN-like lesions with monotypic light chain restriction, most commonly IgG3 with restricted kappa or lambda expression.4 In our case, immunofluorescence on eight glomeruli showed both kappa (1+) and lambda (2+) positivity alongside IgG (1+) and IgM (2+), indicating a polyclonal immune complex pattern. This finding, supported by electron microscopy demonstrating subendothelial and mesangial deposits without organized structures, argues against PGNMID. However, several caveats must be emphasized.

First, the kidney biopsy sample was limited, with light microscopy evaluating only one non-sclerotic glomerulus; focal monotypic deposits might have been missed due to sampling error. Second, pronase-digested paraffin immunofluorescence was not performed. This technique can unmask hidden deposits and should be considered standard for suspected MGRS (9). Third, the possibility that monoclonal IgM participated in immune complex formation cannot be entirely excluded. Therefore, MGRS could not be definitively excluded.

The absence of pronase-digested paraffin immunofluorescence in this case represents a methodological limitation that reduces diagnostic certainty. We have retrospectively confirmed that no additional paraffin blocks or frozen tissue remain available for this procedure. Consequently, the distinction between coincident MGUS and true MGRS cannot be resolved in this patient. This diagnostic uncertainty is the central teaching point: in the absence of gold-standard pathologic workup, clinicians must make treatment decisions recognizing that the classification remains provisional.

The following diagnostic questions remain unanswered in this case:

Were masked monotypic deposits present? (Cannot be determined).

Could this represent PGNMID with sampling error? (Cannot be excluded).

Can MGRS be definitively ruled out? (No).

We propose that similar cases should undergo the following standardized pathologic workup: (1) fresh tissue immunofluorescence with comprehensive immunoglobulin and light chain staining; (2) paraffin immunofluorescence after pronase digestion to detect masked monotypic deposits; (3) electron microscopy with systematic search for organized deposits; and (4) if initial sampling is inadequate, consideration of repeat biopsy. Failure to complete this workflow risks misclassification and inappropriate treatment selection.

### Clinical context and the MGRS suspicion index

Although MGUS may be an incidental finding in older individuals, the combination of nephrotic-range proteinuria, AKI, hypocomplementemia, an IgM-κ monoclonal gammopathy, and a markedly abnormal free light chain ratio (11.224, reference 0.26–1.65) raises a legitimate suspicion that the clone could be involved in the kidney disease. This clinical constellation—while not pathognomonic for MGRS—deserves careful consideration even when immunofluorescence appears polyclonal. The free light chain ratio abnormality, in particular, suggests ongoing clonal activity that may contribute to immune complex formation or complement dysregulation. We acknowledge that without definitive pathologic evidence of monoclonal immunoglobulin deposition in the kidney, this remains speculative; however, the clinical context should not be dismissed solely on the basis of a single limited biopsy.

### Differential diagnosis: cryoglobulinemic glomerulonephritis

The pathologist's observation of pseudothrombi raised consideration of cryoglobulinemic glomerulonephritis, which was relevant given the IgM-κ monoclonal protein, low C3, and low-normal C4. Type II cryoglobulinemia, characterized by monoclonal IgM with rheumatoid factor activity, is associated with hepatitis C infection but can also occur in monoclonal gammopathies (10). However, cryoglobulin testing was negative on three occasions under strict 37°C conditions, hepatitis C RNA was negative, extrarenal manifestations were absent, and electron microscopy showed no organized microtubular structures. These findings make cryoglobulinemic glomerulonephritis unlikely, though not absolutely excluded. Notably, rheumatoid factor was not measured, which remains a diagnostic limitation.

### Complement analysis

The low serum C3 (0.59 g/L) with low-normal C4 (0.10 g/L) suggests classical pathway complement activation, typical of immune complex-mediated diseases.^1^ However, without CH50, C1q, or alternative pathway factor measurements, we cannot definitively characterize the complement activation pathway or exclude secondary alternative pathway involvement. We recommend that similar cases include at least CH50 and C1q testing to better define complement involvement.

### Treatment attribution and follow-up limitations

The patient showed a favorable early response after methylprednisolone pulse therapy followed by rituximab. At 1-month follow-up, kidney function, proteinuria, and serum albumin improved substantially, and complement levels normalized. However, causality cannot be attributed to rituximab from this single case. High-dose corticosteroids were administered simultaneously; supportive care and spontaneous recovery from acute injury may have contributed. Moreover, because the rituximab induction course was ongoing at the time of writing and CD19/CD20 B-cell monitoring had not yet been performed, we cannot confirm successful B-cell depletion or distinguish the respective contributions of corticosteroids and rituximab. Therefore, the observed response should be considered hypothesis-generating rather than proof of treatment efficacy. Future similar cases should consider delayed rituximab initiation or sequential monitoring of B-cell counts to better attribute therapeutic effects.

The rituximab dose of 375 mg/m^2^ (approximately 500 mg for this patient) follows the standard lymphoma protocol, administered weekly for four doses. This differs from some reported MGRS regimens but represents an established dosing framework for B-cell depletion (7). The optimal dosing for IC-MPGN with coincident MGUS remains undefined, and early relapse remains a possibility if B-cell depletion is incomplete. Long-term follow-up of at least 6–12 months is needed to assess sustained remission and clone evolution. The short follow-up period (one month) represents a significant limitation; key outcomes remain unknown, including sustained remission duration, clone evolution (progression to lymphoplasmacytic lymphoma or Waldenström macrogulinemia), long-term kidney function trajectory, and optimal rituximab maintenance strategy. We intend to report subsequent outcomes as they become available.

### MGUS prevalence and clinical context

MGUS is present in approximately 3% of individuals aged 50 years or older and in more than 5% of those aged 70 years or older (11). In a 60-year-old patient with incidentally discovered kidney disease, the probability of MGUS and kidney disease occurring by chance is non-negligible (12). Without definitive pathologic evidence of monoclonal immunoglobulin deposition in the kidney, the possibility that the MGUS is an unrelated coincidental finding cannot be excluded. This probabilistic consideration reinforces the need for comprehensive pathologic workup in all cases of suspected MGRS.

The management of this patient required collaboration among gastroenterology, nephrology, hematology, and pathology. This multidisciplinary approach was essential for navigating the diagnostic uncertainties.

### Patient perspective

The patient stated: “At first I thought I just had indigestion and heartburn, and never expected it to be a kidney and blood problem. After the treatment, the swelling went down completely, my appetite came back, and I felt much better. The doctors told me to have regular check-ups, and I will follow up as advised.”

## Data Availability

The original contributions presented in the study are included in the article/supplementary material, further inquiries can be directed to the corresponding author.
